# Comparison of general anesthesia and deep sedation for complex atrial ablation procedures using a lattice-tip catheter with monopolar pulsed field and radiofrequency ablation

**DOI:** 10.1007/s10840-026-02397-5

**Published:** 2026-07-07

**Authors:** Theresa Storz, Thomas Kueffer, Gregor Thalmann, Corinne Jufer, Valon Spahiu, Claudia Herrera, Boldizsar Kovacs, Nikola A. Kozhuharov, Helge Servatius, Andreas Haeberlin, Fabian Noti, Hildegard Tanner, Laurent Roten, Tobias Reichlin

**Affiliations:** 1 Department of Cardiology, Inselspital, Bern University Hospital, University of Bern, Freiburgstrasse 18, Bern, 3010 Switzerland; 2 SITEM Center for Translational Medicine and Biomedical Entrepreneurship, University of Bern, Bern, Switzerland

**Keywords:** Atrial fibrillation, Pulsed field ablation, General anesthesia, Deep sedation, Lattice-tip catheter

## Abstract

**Background:**

A dual-energy lattice-tip catheter with full 3D integration using monopolar pulsed field ablation (PFA) can result in significant skeletal muscle recruitment. It has therefore mainly been utilized under general anesthesia (GA), but procedures might also be possible using propofol-based deep sedation (DS).

**Methods:**

Consecutive patients undergoing ablation of atrial fibrillation or left atrial flutter using the lattice-tip catheter were enrolled. The use of PFA and/or RFA was based on anatomical location. Procedural characteristics and outcomes were compared between patients with GA and those with DS.

**Results:**

A total of 101 patients underwent ablation, 54% were treated under GA and 47% received DS (median age 69 years, 40% women, 85% redo-procedures). The arrhythmia subtypes were atrial fibrillation in 26%, left atrial flutter in 44%, and both in 31%. In addition to PVI, a total of 150 left atrial extra-PV lesion sets were performed, and CTI ablation in 59 patients. Median laboratory occupancy time was significantly shorter in the deep sedation group (GA 203 min [157–231] vs. DS 162 min [IQR 148–196], *p* = 0.003). No conversions from deep sedation to general anesthesia were required. Freedom from atrial arrhythmia recurrence was higher in the GA group (76% vs. 49% in the DS group, *p* = 0.038). After adjustment for confounders, the association attenuated but remained directionally consistent in favor of GA (HR 0.47 for recurrence, *p* = 0.061).

**Conclusions:**

Complex atrial ablation using a dual-energy lattice-tip catheter appears safe under both GA and DS. Despite higher baseline risk, GA patients had fewer arrhythmia recurrences at one year.

**Graphical abstract:**

**Figure:**
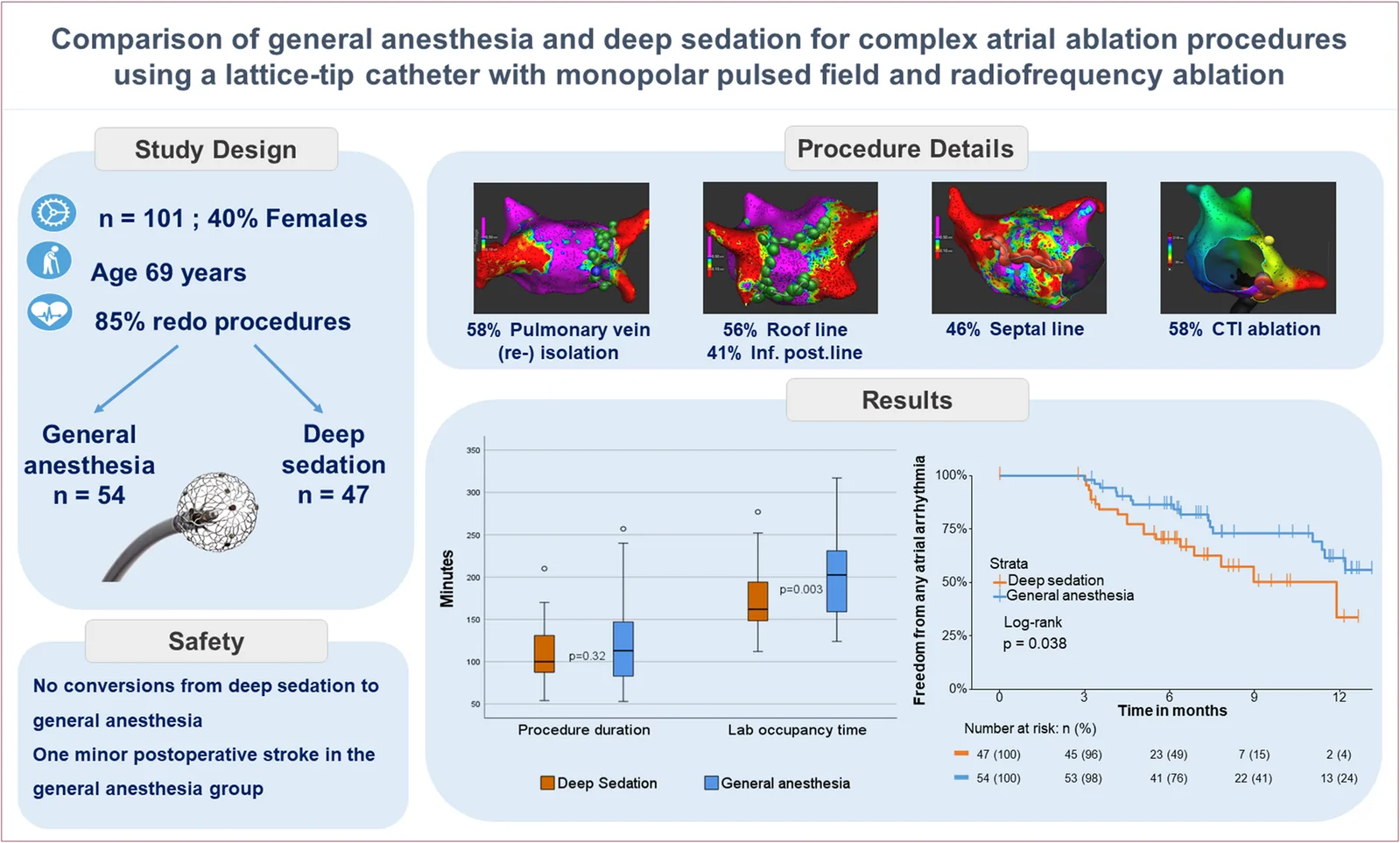

**Supplementary Information:**

The online version contains supplementary material available at https://doi.org/10.1007/s10840-026-02397-5.

## Introduction

Pulsed field ablation (PFA) has emerged as a promising modality for the treatment of atrial fibrillation (AF), characterized by high efficacy and a favorable safety profile compared to thermal ablation [1–4]. Despite the advantages of PFA devices optimized for circumferential pulmonary vein isolation (PVI), their application is often limited by a lack of versatility, particularly when addressing more complex arrhythmogenic substrates beyond the pulmonary veins.

The Affera system (Medtronic) offers a potential solution to this limitation by integrating high-density mapping with a versatile large area focal lattice-tip catheter capable of delivering both monopolar pulsed field and radiofrequency energy [5, 6]. This could be particularly advantageous for patients with persistent AF and atypical left atrial flutter, where mapping is needed to identify the relevant substrates and ablation strategies beyond PVI are often required [5, 7].

Pulsed field ablation (PFA) can cause significant pain and discomfort. In early clinical studies, PFA was predominantly performed under general anesthesia with endotracheal intubation and muscle relaxation [8]. Most currently approved PFA systems employ bipolar PFA, applying localized energy between electrodes of the same catheter. For most of those, deep sedation is a suitable alternative to general anesthesia [9–11]. Monopolar PFA, delivering energy between the catheter and a dispersive patch on the body surface, has the potential to create deeper lesions, but results in more pronounced recruitment of skeletal musculature, which may result in catheter instability or map-shifts due to movement. For monopolar PFA, there is a paucity of scientific data regarding the use of deep sedation for monopolar PFA procedures [12], which has typically been performed under general anesthesia [5, 6].

The aim of our study therefore was to compare the safety and efficacy of performing complex atrial ablations with a monopolar PFA system under either general anesthesia or deep sedation.

## Methods

### Study population

Consecutive patients who underwent left atrial ablation using the AFFERA system at the Inselspital, Bern University Hospital, Bern, Switzerland between May 2024 and May 2025 were prospectively enrolled in this study. All participants provided written informed consent. The registry was approved by the local ethics committee (approval number PB_2018 − 00226), and the study was carried out in accordance with the principles of the Declaration of Helsinki. The authors had full access to and take full responsibility for the integrity of the data.

### Pre-procedural examinations and periprocedural management

Prior to the procedure, patients underwent transesophageal echocardiography and/or cardiac computed tomography to exclude intracardiac thrombi and to assess left atrial anatomy.

Early cases, as well as high-risk patients, received general anesthesia including muscle relaxants because of the anticipated skeletal-muscle activation caused by monopolar PFA. Institutional criteria for the use of general anesthesia included anticipated respiratory difficulties (such as OSA or severe pulmonary disease), obesity, and anticipated hemodynamic difficulties. The remaining procedures were performed using a deep sedation protocol with midazolam, fentanyl and propofol delivered by nurses and supervised by the operating physician [7, 13].

Left atrial access was obtained by fluoroscopy-guided transseptal puncture using either a standard sheath or by direct puncture through an Agilis sheath (Abbott, IL, USA) using a long needle (BRK, 98 cm, Abbott, IL, USA). Intravenous heparin was administered to maintain an activated clotting time above 300 s before placing left atrial catheters and during the procedure. To mitigate the vagal response to ablation, 0.5 mg of atropine was administered immediately before the first ablation.

### Ablation system and PVI procedure

The AFFERA System pairs a dual-energy, 9 mm lattice-tip catheter with nine miniature mapping electrodes and nine temperature sensors, providing high-density 3D electroanatomical mapping (3D-EAM) and the ability to deliver both temperature-controlled radiofrequency (RF) and pulsed-field (PF) energy. The system is described elsewhere in detail [5]. The “Pulse3” (4 s) preset was used for all PF lesions, whereas RF energy was delivered using the “Antral” (5 s) protocol. Tissue contact was assessed by impedance change, and lesion quality assessed based on the temperature rise during the ablations. No intracardiac echocardiography was employed.

For PVI, radiofrequency ablation was confined to the anterior aspects, whereas pulsed field ablation was used for the posterior aspects to reduce the risk of esophageal injury. Roof and inferior lines were created with PF, and anterior and posterior mitral-isthmus lines were produced with sequential RF followed by PF to avoid electrogram stunning before verifying bidirectional block. Superior vena cava isolation employed a mixed RF/PF strategy after mapping the sinus node and confirming the absence of phrenic nerve capture. Cavotricuspid-isthmus (CTI) ablation was performed exclusively with RFA to avoid coronary spasms. Completeness of each lesion set was confirmed by post-ablation mapping, exit pacing for PVs, and demonstration of bidirectional block across linear lesions.

### Procedural characteristics

Procedure time was measured from groin access to removal of the sheaths. Lab occupancy time was measured from arrival in to departure from the electrophysiology laboratory. The safety endpoints included the need for conversion from deep sedation to general anesthesia, as well as, procedural complications requiring intervention during catheter ablation, the time until discharge as well as the following 30 days.

### Follow-up

Outpatient visits - including a 7-day electrocardiogram (ECG) - were scheduled at 3, 6, and 12 months. The primary efficacy endpoint was recurrence of any atrial tachyarrhythmia (atrial fibrillation, atrial flutter or atrial tachycardia), lasting > 30 s without a 90-day blanking period. The safety endpoint comprised a composite of in-hospital major adverse events. Lesion durability data was collected during left-atrial repeat procedures.

### Statistical analysis

Continuous variables are reported as mean ± standard deviation or median (interquartile range), as appropriate. Freedom from arrhythmia recurrence was estimated with the Kaplan-Meier method and compared using the log-rank test. Categorical variables were analyzed with Fisher’s exact test, and continuous variables with the Mann-Whitney-U-test. Baseline characteristics were examined using Cox proportional hazards models to predict recurrence of any atrial tachyarrhythmia. Variables with a p-value < 0.1 were selected for the multivariable model. Multiple imputations were performed to account for missing data. Lesion durability was compared using lesion-level logistic regression with cluster-robust standard errors. All tests were two-sided, and a p-value < 0.05 was considered statistically significant. Statistical analyses were performed using SPSS V28.0.1.1 (14) (IBM, Armonk, NY, USA) and R 4.5.0 (R Core team, Vienna, Austria).

## Results

### Baseline characteristics

A total of 101 patients were included in the analysis. 54 patients (54%) underwent general anesthesia with endotracheal intubation, while 47 patients (47%) received propofol-based deep sedation. Of the population, 40% were female and 85% underwent redo procedures, 41 (76%) in the GA group and 45 (96%) in the DS group (*p* = 0.005, Table 1). The arrhythmia subtypes comprised of atrial fibrillation in 26% cases, atypical left atrial flutter in 44%, and a combination of both in 31%.

**Table 1:** Characteristics of the patients at baseline*

	All patients (*n* = 101)	General anesthesia (*n* = 54)	Deep sedation (*n* = 47)	*P*-value
Age, years	69 (62–76)	67 (56–73)	73 (68–78)	0.002
Female Sex, no.%	40 (39.6)	15 (27.8)	25 (53.2)	0.014
Body mass index, kg/m^2^	28 (25–32)	29 (26–35)	26 (25–30)	0.003
CHADS-VA Score §	2 (1–3)	2 (1–3)	2 (2–3)	0.56
Concomitant clinical conditions, no. (%)				
Hypertension, %	62 (61.4)	34 (63.0)	28 (59.6)	0.84
Diabetes, %	12 (11.9)	9 (16.7)	3 (6.4)	0.13
Previous stroke or TIA, %	18 (17.8)	11 (20.4)	7 (14.9)	0.60
Obstructive Sleep Apnea Syndrome, %	18 (17.8)	15 (27.8)	3 (6.4)	0.008
Echocardiographic data				
LVEF, %	55 (49–60)	55 (46–60)	55 (50–55)	0.80
LA volume index (ml/m^2^)	42 (35–51)	45 (36–54)	41 (35–48)	0.28

Numbers are median (IQR) or counts (%)

*TIA* transient ischemic attack, *LVEF*  left ventricular ejection fraction, *LA*  left atrial

§ CHA2DS2-VA score (an assessment of the risk of stroke among patients with atrial fibrillation) range from 0 to 8, with higher scores indicating a higher risk of stroke

* Details of preablation treatment with antiarrhythmic drugs are provided in Table S1

### Procedural data

**Fig. 1:**
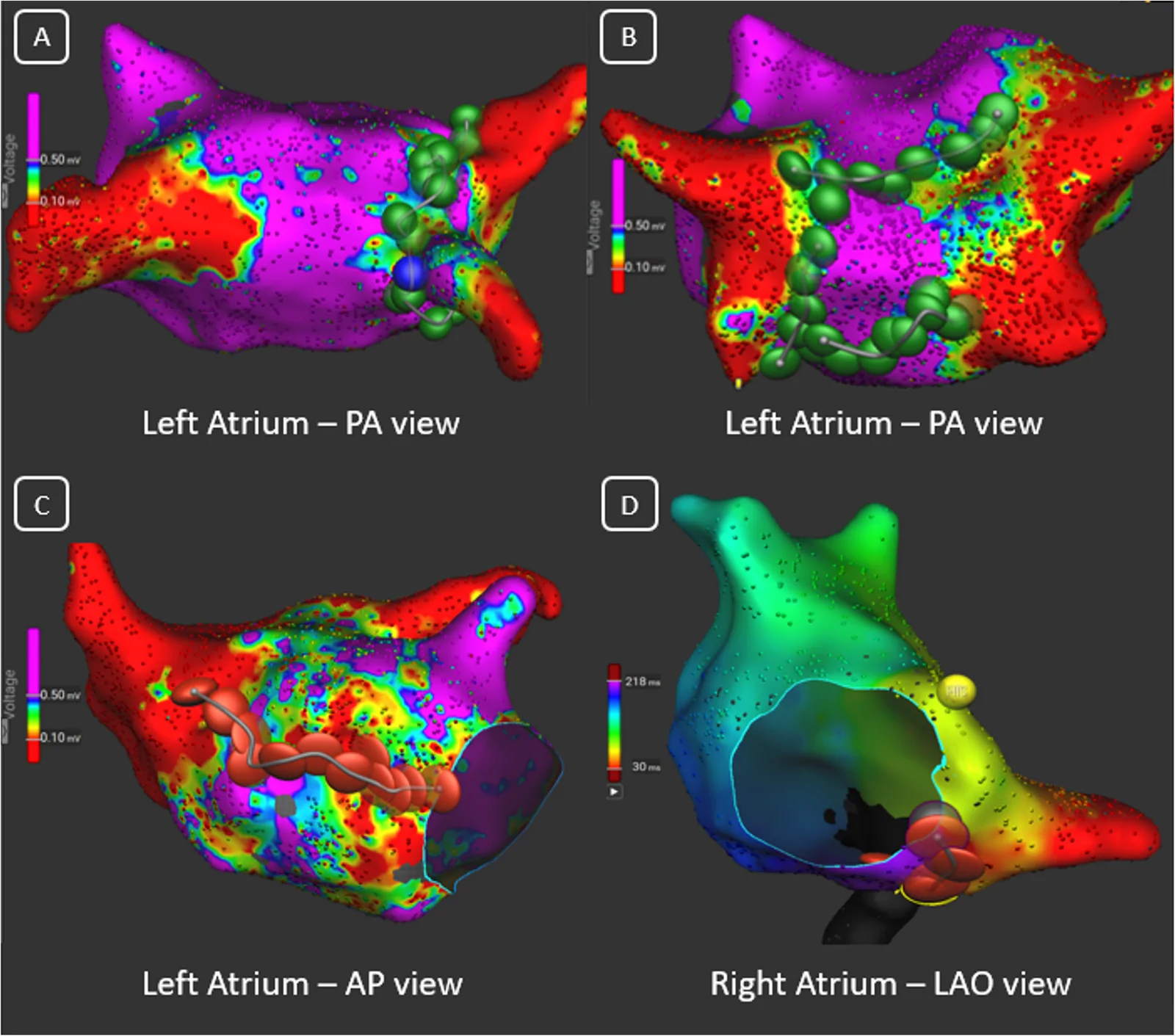
Selection of ablation lesion sets. Three-dimensional bipolar voltage maps showing a selection of the lesion sets performed: Re-Isolation of reconnected pulmonary veins (Panel **A**), posterior wall ablation by means of a superior and inferior posterior line (Panel **B**), a septal line connecting the anterior mitral annulus to the right upper pulmonary vein (Panel **C**) and cavotricuspid isthmus ablation (Panel **D**). Green dots mark PF lesions and red dots mark RF lesions. Around the ipsilateral PVs as applied by the lattice-tip mapping and ablation catheter

High-density voltage mapping was successfully completed in all patients at the outset of the procedure. Pulmonary vein (re-)isolation was performed in 29 patients (54%) of the general anesthesia cohort and in 30 patients (64%) of the deep sedation cohort. Beyond pulmonary vein isolation, 70 extra-PV lesion sets were performed in the general anesthesia group (25 roof lines, 18 inferior posterior lines, 27 septal lines), and 80 extra-PV lesion sets were performed in the deep sedation group (32 roof lines, 23 inferior posterior lines, 25 septal lines; Table 2; Fig. 1). Cavotricuspid isthmus ablation was added in 31 patients (57%) in the general anesthesia group and 28 patients (60%) in the deep sedation group.

**Table 2:** Procedural characteristics

	All patients (*n* = 101)	General anesthesia (*n* = 54)	Deep sedation (*n* = 47)	*P*-value
Lesion sets				
Pulmonary vein (Re-)Isolation	59 (58.4)	29 (53.7)	30 (63.8)	0.32
Roof line*	57 (56.4)	25 (46.3)	32 (68.1)	0.04
Inferior posterior line^§^	41 (40.6)	18 (33.3)	23 (48.9)	0.16
Septal line^#^	52 (51.4)	27 (50)	25 (53.1)	0.75
Mitral isthmus line^†^	4 (4.0)	3 (5.6)	1 (2.1)	0.71
Cavotricuspid isthmus line	59 (58.4)	31 (57.4)	28 (59.6)	0.83
Repeat procedure	86 (85.1)	41 (75.9)	45 (95.7)	0.005
Procedure duration (min)	105 (85–143)	113 (83–147)	100 (88-131-)	0.32
Fluoroscopy time (min)	8.0 (4–11)	7.0 (4–11)	8.0 (5–11)	0.32
Fluoroscopy dose (cG*cm^2^)	457 (183–1574)	525 (184–1840)	433 (188–934)	0.26
Lab occupancy time (min)	173 (150–216)	203 (157–231)	162 (148–196)	0.003

Numbers are median (IQR) or counts (%)

* Connecting the superior aspect of the right superior and left superior pulmonary vein

§ Connecting the inferior aspect of the right inferior and left inferior pulmonary vein

# Connecting the mitral annulus to the right superior pulmonary vein

† Connecting the mitral annulus to the left inferior pulmonary vein

**Fig. 2:**
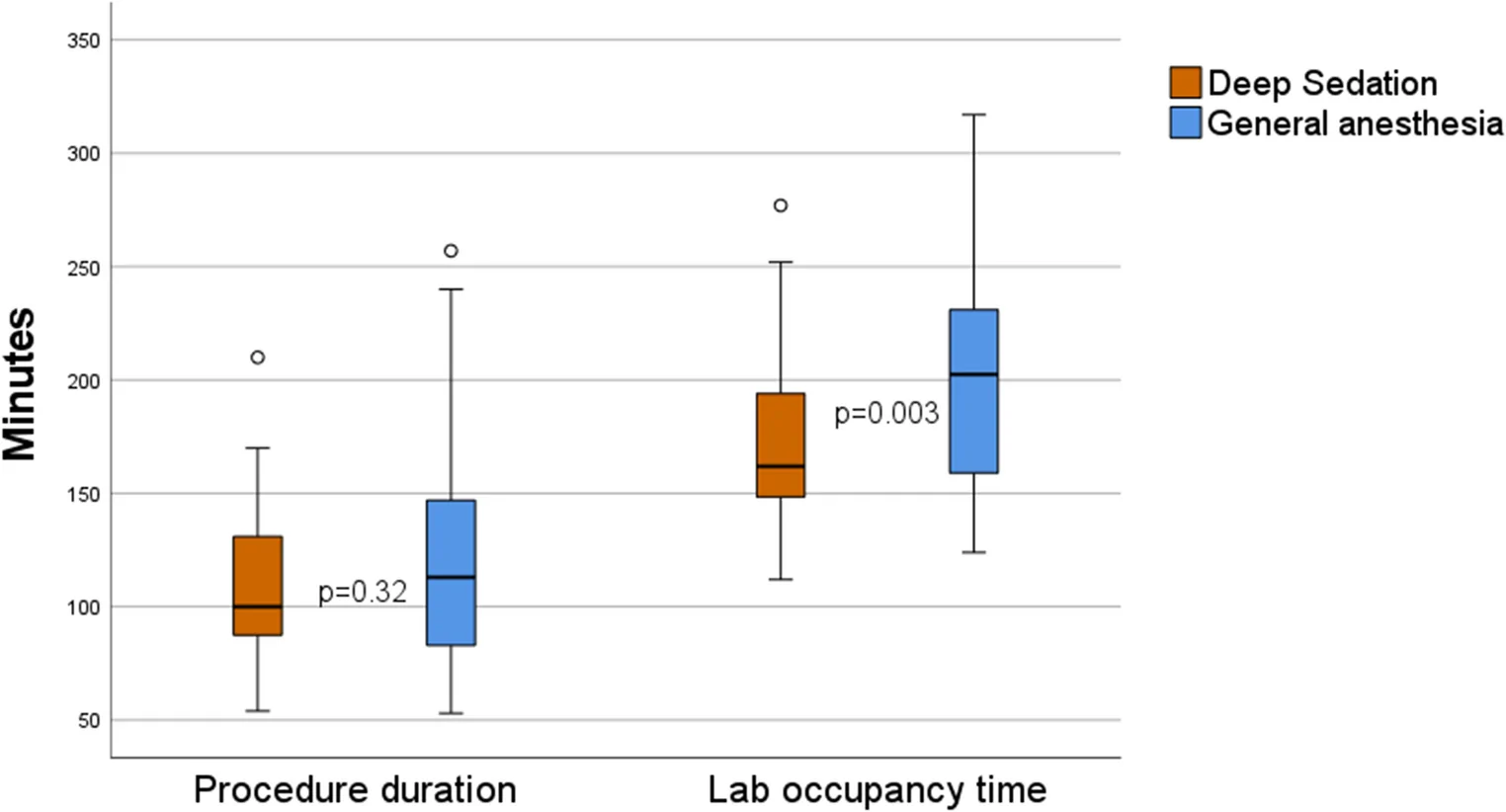
Procedure duration and lab occupancy time. Boxplots indicating total procedure time (left panel) and total lab occupancy time (right panel). Each box represents the interquartile range, with the horizontal line inside indicating the median

Median procedure time was similar between the two groups (general anesthesia 113 min [IQR 83–147] vs. deep sedation 101 min [IQR 85–133], *p* = 0.32). Laboratory occupancy time, however, was significantly shorter in the deep sedation group (median 162 min, IQR 148–196) compared to the general anesthesia group (median 203 min, IQR 157–231, *p* = 0.003), Fig. 2. The mean fluoroscopy time was 7.0 min in the general anesthesia group and 8.0 min in the deep sedation group (*p* = 0.32).

### Safety

No conversions from deep sedation to general anesthesia were required. One patient within the general anesthesia group experienced a minor ischemic stroke, presenting post-procedurally with torsional vertigo. Emergency diffusion-weighted MRI demonstrated an acute, punctate ischemic lesion in the left cerebellum. Complete resolution of symptoms occurred within one night. No cases of pericardial tamponade, vascular complications, or phrenic nerve paralysis were observed in either group.

### Outcomes

**Fig. 3:**
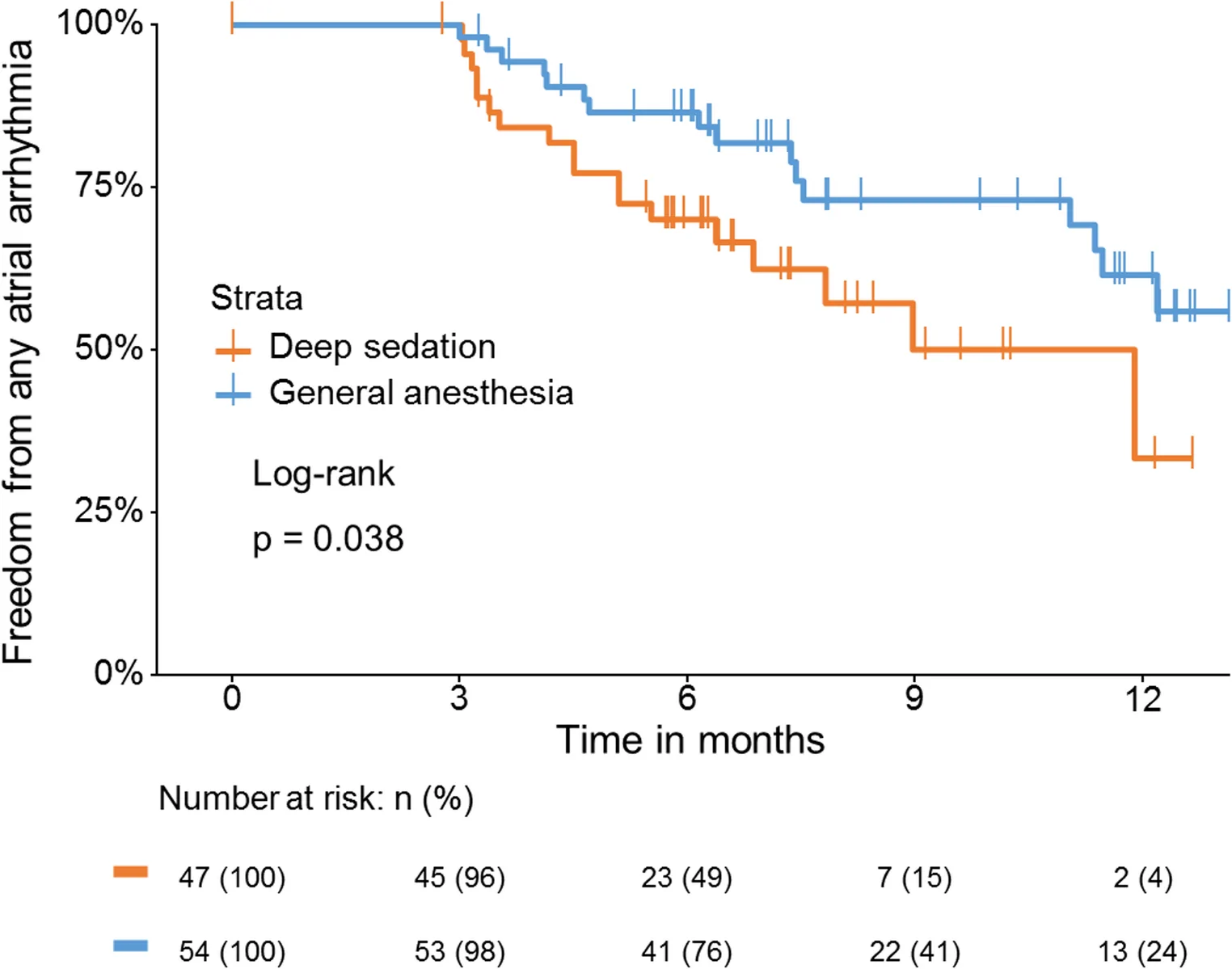
Recurrence of atrial arrhythmias during follow-up. Kaplan Meier curve showing freedom from recurrence of atrial tachyarrhythmia up to 12 months

At the end of the procedure, all PVs were isolated and bidirectional block was achieved for all left and right atrial linear lesions. At one year, patients in the general anesthesia group showed higher freedom of atrial arrhythmia compared to deep sedation (62% vs. 33%, *p* = 0.038, Fig. 3). After adjusting for potential confounders identified in univariable Cox proportional hazards analyses, general anesthesia (vs. deep sedation) showed a trend to fewer recurrences with a hazard ratio of 0.45, CI 0.22–1.13, *p* = 0.061 (Table 3).

**Table 3:** Factors associated with recurrence of any atrial arrhythmia

*N* = 101 patients	Univariable			Multivariable		
Characteristic	HR	95% CI	*p*-value	HR	95% CI	*p*-value
Male sex	0.97	0.49, 1.91	0.93			
Non-paroxysmal AF	1.81	0.86, 3.81	0.12	2.31	1.01, 5.30	0.048
Atypical flutter during case	0.71	0.36, 1.41	0.33			
General anesthesia	0.48	0.24, 0.97	0.041	0.50	0.22, 1.13	0.091
Age	1.02	0.99, 1.05	0.30			
BMI	1.02	0.95, 1.10	0.53			
CHA2DS2-Va	0.94	0.74, 1.19	0.60			
Left-atrial volume index	1.01	0.98, 1.03	0.60			
LVEF	1.01	0.97, 1.04	0.71			
Procedure duration	1.00	0.99, 1.01	0.63			
Repeat procedure	7.80	1.06, 57.2	0.043	5.50	0.66, 45.8	0.11
Operator volume > 200 cases per year	0.48	0.22, 1.05	0.068	0.51	0.22, 1.18	0.11

Cox proportional hazards models to predict recurrence of any atrial tachyarrhythmia. Variables with a *p*-value < 0.1 were selected for the multivariable model

CHA2DS2-VA score (an assessment of the risk of stroke among patients with atrial fibrillation) range from 0 to 8, with higher scores indicating a higher risk of stroke

*CI* Confindence Interval, *HR* Hazard Ratio, *LVEF*  left ventricular ejection fraction

Fifteen patients underwent left atrial repeat ablation, including 10 (19%) after general anesthesia and 5 (11%) after deep sedation (*p* = 0.41). Two additional patients underwent AV node ablation. Among 33 evaluable lesions, durability was observed in 15/24 (63%) lesions after general anesthesia and 6/9 (67%) after deep sedation. In clustered lesion-level analysis, durability did not differ between groups (OR 0.83, 95% CI 0.22–3.15, *p* = 0.79).

## Discussion

This prospective study of 101 consecutive patients undergoing ablation of left atrial arrhythmias using a dual-energy large-area lattice-tip catheter is the largest cohort to date reporting on the anesthesia aspects associated with the use of this system. Furthermore, this is the first study on the subject that shows not only acute success rates but also follow-up and durability data. Our study provides important insights for the treatment of those patients, and we report three major findings:

First, monopolar PFA was feasible under both deep sedation and general anesthesia in a complex ablation cohort, with extra-PV lesions performed in 78% of patients and no safety events observed.

Second, procedure duration and fluoroscopy time were similar, but lab occupancy time was significantly shorter in the deep sedation group (162 vs. 203 min).

Third and probably most importantly, despite higher baseline risk and achieving acute success all patients, the use of general anesthesia resulted in lower recurrence rates compared to the used of deep sedation.

There is only limited evidence on the use of the Affera system under deep sedation. Rillig et al. investigated a cohort of 63 patients treated with the Affera system (40 deep sedation, 23 general anesthesia). In the vast majority of cases, only PVI was performed, which is a major difference to our patient cohort. They reported a significantly shorter laboratory occupancy time in the deep sedation group but no difference in the procedure duration, which is consistent with our observations. Pulmonary vein isolation (PVI) was acutely successful in 100% of patients. However, no long-term outcome data were provided in their report. They concluded that the system can be safely and effectively used under deep sedation [12]. Our findings also confirm that the use of the Affera system under deep sedation is safe and feasible. However, follow-up data suggest an advantage for the general anesthesia group despite acute success in both groups. The relatively low absolute arrhythmia-free survival reflects the complexity of the cohort: 85% were redo cases, often multiply-pretreated and referred for refractory arrhythmias, in whom recurrence rates are inherently high.

A possible explanation for the reduced effectiveness of deep sedation is increased patient and catheter movement during monompolar PFA applications used in the lattice-tip catheter, which has been associated with greater skeletal muscle recruitment [14]. Increased movement may destabilize catheter position during energy delivery, impair catheter–tissue coupling, and lead to suboptimal lesion formation. However, repeat-procedure data in the present cohort did not demonstrate a difference in lesion durability between groups, and this hypothesis remains speculative.

Our findings have clinical implications: General anesthesia adds procedural complexity, often leading to longer laboratory occupancy and higher costs. Currently, the vast majority of PFA procedures in Europe use bipolar PFA and are carried out under deep or conscious sedation [9, 15, 16]. Should evidence solidify that deep sedation is associated with worse arrhythmia control, the use of deep sedation rather than GA should be reconsidered for lattice tip catheter cases. As the sphere-9 catheter represents one of the first commercially available monopolar PFA systems, further waveform optimizations may enable monopolar PFA with minimal muscle recruitment [17]. Muscle relaxants would eliminate patient movement, but general anesthesia and mechanical ventilation are required for their use [5, 18].

Procedure durations did not differ between groups but procedures performed under deep sedation were associated with shorter laboratory occupancy times - a finding of economical and even more clinical relevance given the limited electrophysiology lab capacity in many centers and the growing number of patients with atrial fibrillation [19]. However, while shorter turnover times may improve workflow efficiency, the long‑term clinical benefit for patients ultimately depends on arrhythmia control. From both a patient‑centered and socioeconomic perspective, higher recurrence rates are undesirable, as each recurrence is associated with increased healthcare utilization, repeat procedures, and overall costs.

### Patient selection

Patients undergoing GA procedures are selected because of an increased risk of deep sedation-related complications. Accordingly, in our GA cohort, patients were older, had higher BMI, and more frequently had OSAS. Some of these factors, particularly age and BMI, have been associated with worse arrhythmia outcomes in larger cohorts. One might therefore expect worse outcomes in the GA group.

Despite this higher baseline risk, GA patients experienced fewer recurrences, an effect that remained directionally consistent after adjustment for confounders although the limited sample size precludes firm conclusions (HR for recurrences 0.47, *p* = 0.061). Procedural effectiveness was similar between groups (100% PVI and bidirectional block across all extra-PV lines), arguing against inadequate ablation as an explanation for the poorer outcomes observed in the DS group.

Operator experience is a further potential source of bias, as general anesthesia was used preferentially for the earliest cases. Grouping operators by annual volume (cutoff 200 cases/year) showed no significant association with recurrence, and the anesthesia effect was unchanged, though a residual learning-curve effect cannot be excluded.

### Limitations

Our study has several limitations that should be considered when interpreting our findings. First, this was a single-center study from a center with substantial experience using deep sedation. Replication of our findings from other centers will be insightful. Second, this is a non-randomized prospective study that may carry residual bias even after careful adjustment for confounders. Third, patient and operator satisfaction was not prospectively assessed in our study. However, no negative patient feedback was reported in both groups. And finally, our study employed a propofol-based deep sedation protocol. Although a recent randomized controlled trial has demonstrated the suitability of a remimazolam–ketamine protocol for deep sedation with a bipolar pentaspline PFA system, its application with the lattice-tip monopolar PFA system has not yet been investigated.

## Conclusions

Complex atrial ablation with a dual‑energy lattice‑tip catheter using monopolar PFA was safe and feasible under both general anesthesia and deep sedation. Despite higher baseline risk, GA patients had fewer arrhythmia recurrence at one year, suggesting improved procedural outcomes that may reflect compromised catheter-tissue coupling under deep sedation due to patient movement.

## Electronic Supplementary Material

Below is the link to the electronic supplementary material.

Supplementary Material 1 (DOCX 13.6 KB)

## Data Availability

The Affera case database used in this study was established within the framework of the current ethics application. These data form the basis of the analyses presented here and are not publicly available due to restrictions related to patient confidentiality and ethical approval, but may be made available upon reasonable request and with the approval of the responsible institutional or data‑governing body.

## Abbreviations

AF: atrial fibrillation
AFL: atrial flutter
BP: blanking period
CTI: cavotricuspidal isthmus
DS: deep sedation
ECG: electrocardiogram
GA: general anesthesia
LA: left atrium/atrial
LVEF: left ventricular ejection fraction
PV: pulmonary vein
PVI: pulmonary vein isolation
PFA: pulsed field ablation
RFA: radio frequency ablation
