# Supplementary material for: Comparison of general anesthesia and deep sedation for complex atrial ablation procedures using a lattice-tip catheter with monopolar pulsed field and radiofrequency ablation
Source: J Interv Card Electrophysiol. 2026 Jul 7;69(9):2861–9. doi: 10.1007/s10840-026-02397-5 (PMC13769683; doi:10.1007/s10840-026-02397-5)
Supplement: Supplementary file 1 [file 10840_2026_2397_MOESM1_ESM.docx]

|  | **All Patients**  (n=101) | **General Anesthesia**  (n=54) | **Deep Sedation**  (n=47) | **P-Value** |
| --- | --- | --- | --- | --- |
| Class 1 | 3 (3.0) | 1 (1.0) | 2 (2.0) | 0.480 |
| Class 2 | 76 (75.2) | 42 (41.6) | 34 (33.7) | 0.530 |
| Class 3 | 11 (10.9) | 1 (1.0) | 10 (9.9) | 0.009 |
| Class 4 | 2 (2.0) | 0 | 2 (2.0) | 0.185 |
|  |  |  |  |  |
| Class 5 | 0 | 0 | 0 | 1.00 |

S1 Preablation treatment with antiarrhythmic drugs

Numbers are counts (%).
